# Interleukin-1 receptor antagonist delivered directly and by gene therapy inhibits matrix degradation in the intact degenerate human intervertebral disc: an *in situ *zymographic and gene therapy study

**DOI:** 10.1186/ar2282

**Published:** 2007-08-30

**Authors:** Christine L Le Maitre, Judith A Hoyland, Anthony J Freemont

**Affiliations:** 1 Tissue Injury and Repair Group, Research School of Clinical and Laboratory Sciences, The School of Medicine, University of Manchester, Manchester M13 9PT, UK

## Abstract

Data implicate IL-1 in the altered matrix biology that characterizes human intervertebral disc (IVD) degeneration. In the current study we investigated the enzymic mechanism by which IL-1 induces matrix degradation in degeneration of the human IVD, and whether the IL-1 inhibitor IL-1 receptor antagonist (IL-1Ra) will inhibit degradation. A combination of *in situ *zymography (ISZ) and immunohistochemistry was used to examine the effects of IL-1 and IL-1Ra on matrix degradation and metal-dependent protease (MDP) expression in explants of non-degenerate and degenerate human IVDs. ISZ employed three substrates (gelatin, collagen, casein) and different challenges (IL-1β, IL-1Ra and enzyme inhibitors). Immunohistochemistry was undertaken for MDPs. In addition, IL-1Ra was introduced into degenerate IVD explants using genetically engineered constructs. The novel findings from this study are: IL-1Ra delivered directly onto explants of degenerate IVDs eliminates matrix degradation as assessed by multi-substrate ISZ; there is a direct relationship between matrix degradation assessed by ISZ and MDP expression defined by immunohistochemistry; single injections of IVD cells engineered to over-express IL-1Ra significantly inhibit MDP expression for two weeks. Our findings show that IL-1 is a key cytokine driving matrix degradation in the degenerate IVD. Furthermore, IL-1Ra delivered directly or by gene therapy inhibits IVD matrix degradation. IL-1Ra could be used therapeutically to inhibit degeneration of the IVD.

## Introduction

Chronic low back pain either alone or in association with sciatica (CLBP), is a common musculoskeletal disorder causing considerable population morbidity (6% prevalence) and an £11 billion pound annual cost to the UK economy through social and healthcare expenditure and loss of work. Recent controlled studies have established a causal association between degeneration of the intervertebral disc (DIVD) and CLBP [1,2].

Individual intervertebral discs (IVDs) are part of a complex of interdependent spinal structures known as the 'motion segment', in which IVDs facilitate movement and maintain optimal separation and orientation of other elements. This is achieved by a biomechanical balance between the IVD's two main structural elements, the nucleus pulposus (NP) and the annulus fibrosus (AF). Normal NP consists of type II collagen fibres and proteoglycans, notably aggrecan [3], which form a hydrophilic molecular complex that generates a swelling pressure sufficient to separate adjacent vertebrae. Excessive swelling is resisted by tension in the type I collagen fibre arrays of the AF. The balance between swelling of the NP and tension in the AF ensures optimal separation of adjacent vertebral bodies and efficient biomechanics of the motion segment.

DIVD is a disorder characterized by loss of hydrophilic matrix from the NP, leading to reduced vertebral separation, instability of the motion segment, microtrauma, and disc bulging [4]. Searches for a cause of loss of matrix molecules through largely observational studies of enzyme expression have implicated metal-dependent matrix degrading enzymes (metal-dependent proteases (MDPs) – matrix metalloproteinases (MMPs), metalloproteinases with thrombospondin motifs (ADAMTS)) in matrix degradation in DIVD [3,5-8]. However, not all data completely support this view [7-9], and direct evidence from intact human tissue is sparse.

Whilst upstream events driving enzyme production are largely unknown, the cytokine IL-1 has been implicated [10-13]. Supporting evidence from our own laboratory includes: showing that by comparison with non-degenerate IVD, in DIVD both isoforms of IL-1 (IL-1α/IL-1β), IL-1 receptor (IL-1R1) and IL-1 converting enzyme are over-expressed [14] without corresponding up-regulation of the natural inhibitor of IL-1, interleukin-1 receptor antagonist (IL-1Ra) [14]; and in monolayer and three-dimensional alginate culture, IL-1Ra will down-regulate expression of MDPs by cells from degenerate IVDs [15].

Inhibiting degenerative processes would be a novel approach to managing DIVD, were it possible to identify key molecular targets. In this context an IL-1-driven, MDP-mediated mechanism would be attractive, with inhibitors such as IL-1Ra already in use in rheumatology [16].

In the current study we have addressed the lack of direct evidence for this mechanism in human tissue, believing this to be an essential step in translating current laboratory data into clinical applications, by examining the hypothesis 'matrix degradation in DIVD is inhibited by IL-1Ra'. In addition, recognizing the difficulties involved in delivering therapeutic agents into degenerate IVDs, we have investigated the hypothesis 'gene therapy is a practical way of delivering IL-1Ra into degenerate IVD'.

We have used *in situ *zymography (ISZ) to assess matrix degradation. *In vivo*, enzyme activity is highly regulated at a number of different levels (for example, gene expression and translation, proenzyme activation, enzyme-matrix interactions, endogenous inhibitors, and so on [17]). As such, conventional approaches (for example, enzyme expression, cells in artificial matrices, extraction zymography) that cannot account for the full spectrum of regulatory controls cannot adequately reflect enzyme activity *in vivo*. ISZ [18] approximates more closely the *in vivo *situation than other techniques and is becoming widely used in oncology [19,20] and musculoskeletal research [21,22] for localizing enzyme activity in human and animal tissues. ISZ works only if active, uninhibited enzyme is present, which means enzyme RNA must have been synthesised, translated to pro-enzyme and the pro-enzyme activated. Furthermore, the reaction will proceed only if there is less 'inhibitor potential' than 'enzyme potential', and because the tissue is intact this will include tissue bound inhibitors.

ISZ is specific for the matrix molecule (for example, type II collagen) but not the enzyme. Conventionally, several matrices are selected that cover the spectrum of enzyme activity being investigated. The tissue can be pretreated to examine the effects of putative stimulators/inhibitors of enzyme activity.

In the experiments described here we have examined enzyme activity directed at degradation of collagen type II, gelatin and casein in intact normal and degenerate human AF and NP tissue, both with and without pretreatment with IL-1 and IL-1Ra. We have also examined the same tissue by immunohistochemistry (IHC) for evidence of expression of a limited number of matrix enzymes that are both expressed in degenerate IVDs and are active against the ISZ matrices.

Finally, having found that IL-1Ra affects matrix degradation, we have investigated a novel method that might form the basis of delivery of IL-1Ra into the human degenerate IVD.

## Materials and methods

### Experiment 1: ISZ localization of matrix degrading activity in normal and degenerate IVDs – the effects of IL-1, IL-1Ra and inhibitors of matrix degrading enzymes

#### Source of human IVDs

Non-degenerate and degenerate IVDs were obtained from 18 cadavers within 16 hours of death (Trent Multicenter Research Ethics Committee – 05/MRE04/03) and 13 live patients (Salford and Trafford [01/049] and Central Manchester [C/01/008] Local Research Ethics Committees) (Table 1). All samples were taken with informed consent of the patient or relative. Two to four parallel, sagittally orientated tissue slabs incorporating AF and NP were taken from each case.

**Table 1:** Details of the individuals and the tissue sources used in these studies (CVA = cerebrovascular accident ["stroke"])

Age (years) and sex	Spinal level	Score	Reason excised/cause of death
**Normal tissue: live patients**			
18 M	L4/5	0	Spinal trauma
	L5/S1	1	
24 M	L3/4	1	Spinal trauma
48 F	L3/4	1	Spinal reconstruction for metastatic breast cancer
**Normal tissue: cadaveric**			
59 F	L3/4	1	Massive pulmonary embolism
52 M	L4/5	1	Myocardial infarction
47 M	L3/4	1	Road traffic accident
72 M	L3/4	1	Myocardial infarction
64 M	L5/S1	2	Myocardial infarction
75 F	L4/5	2	CVA
47 F	L4/5	3	Ovarian cancer
58 M	L4/5	3	Myocardial infarct
**Degenerate tissue: live patients**			
51 M	L4/5	7	Disc degeneration
60 M	L4/5	7	Disc degeneration
25 M	L4/5	8	Disc degeneration
45 M	L4/5	8	Disc degeneration
48 M	L4/5	8	Disc degeneration
73 F	L3/4	9	Disc degeneration
61 M	L5/S1	9	Disc degeneration
45 F	L5/S1	9	Disc degeneration
53 M	L4/5	10	Disc degeneration
46 F	L4/5	11	Data not available
**Degenerate tissue: cadaveric**			
49 F	L4/5	5	Data not available
57 F	L4/5	6	Myocardial infarction
64 M	L4/5	7	Myocardial infarction
69 M	L4/5	8	CVA
47 F	L3/4	8	Breast cancer
76 M	L4/5	9	CVA
50 M	L3/4	9	Myocardial infarction
61 F	L5/S1	10	Data not available
28 M	L4/5	11	Road traffic accident
59 M	L4/5	11	Myocardial infarction

F, female; M, male.

#### Tissue processing and histological assessment

One slab was processed for histology, *in situ *hybridization and IHC, using formalin fixation and paraffin embedding [23]. Samples were checked for orientation and inclusion of AF and NP, and scored for degree of degeneration using a published histological 12 point scale [24]. IVDs scoring 4 to 8 were graded 'moderately degenerate' and 9 to 12 'severely degenerate'. Non-radioactive *in situ *hybridization using a poly T probe for polyadenylated mRNA tested cell viability [25].

From 30 IVDs, the other slabs were used to generate 3 tissue blocks measuring 5 × 3 × 3 mm, incorporating AF and NP for ISZ. From 5 IVDs (score 7 to 8), 3 to 6 5 × 5 × 5 mm blocks of NP were taken for gene delivery studies (see experiment 3 below) and 2 IVDs from an 18 year old male were used to isolate NP cells for these studies as described previously [15].

#### *In situ *zymography

Ten non-degenerate (eight cadaveric, two live patient), ten moderately degenerate (five surgical, five cadaveric), and ten severely degenerate (five cadaveric, five surgical) IVDs were studied. One block was incubated for 48 hours at 35°C in TRIS-HCl buffer, and the other two in buffer supplemented with IL-1β (10 ng/ml recombinant human IL-1β (R&D Systems, Abingdon, Oxfordshire, UK)) [15], or IL-1Ra (100 ng/ml (R&D systems)).

Tissue blocks were snap frozen, and 15 μm tissue sections were examined for evidence of gelatinase, caseinase and type II collagenase activity. ISZ was performed as described previously [21] on 36 randomized serial sections from each block. Sections were mounted on slides precoated with gel containing equal quantities of 1% agarose and 1 mg/ml of FITC-labeled gelatin, casein or bovine type II collagen (Sigma, Poole, Dorset, UK) (It is not possible to gel aggrecan and so aggrecanase activity was not tested directly.) Inside a 100% humidity chamber, 2 randomized serial sections were covered by a large, self-supporting drop of liquid consisting of either TRIS-HCl buffer, pH 7.4, alone or supplemented with an enzyme inhibitor (broad spectrum protease inhibitor (BSIP) consisting of TRIS buffer containing (from Sigma) 200 μM phenylmethyl sulphonyl fluoride (PMSF; serine protease inhibitor), 1 μM leupeptin (serine/cysteine protease inhibitor), 100 μM EDTA (MDP inhibitor), and 1 μM pepstatin (aspartate protease inhibitor) or specific inhibitors consisting of one component only). The zymographic reaction was allowed to proceed for 48 hours at 35°C, changing the medium 12 hourly.

At 0 and 48 hours, slides were viewed in a fluorescence image analyzer, the image captured and area of gel digested measured using semi-automated image analysis.

### Experiment 2: localization and semiquantification of matrix degrading enzyme expression in sections used for ISZ

At the end of each ISZ experiment, sections were removed from the slides, fixed in formalin and paraffin embedded for IHC. The thickness of the sections used for ISZ limited the number of sections reliably available for IHC to three. Based on previous expression studies, MMPs expressed in the degenerate IVD and that preferentially degraded the ISZ matrices (MMP3 (caseinase/aggrecanase/gelatinase), MMP7 (gelatinase/aggrecanase), MMP13 (type II collagenase)) were selected for IHC.

IHC, performed as previously published [5], was used to localize MMP3, MMP 7 and MMP 13 in 3 μm paraffin sections. Antibodies were from R&D systems. Anti-MMP7 and -13 antibodies were mouse monoclonals and the anti-MMP3 antibody a goat polyclonal. Chymotrypsin antigen retrieval was required for anti-MMP7. The presence of bound antibody was disclosed using biotinylated rabbit anti-mouse or donkey anti-goat antibodies in conjunction with a streptavidin, biotin, DAB (3,3'-diaminobenzidine tetrahydrochloride) system. In both AF and NP, 100 to 300 cells (determined by sample size and cellularity) were used to assess the percentage of nucleated cells immunopositive for each enzyme.

### Experiment 3: assessment of the effectiveness of a gene delivery system in inhibiting MDP expression in degenerate human IVDs

Three blocks of NP from two degenerate IVDs and six from three were encapsulated in Perspex rings [26] and investigated for the effect of IL-1Ra, delivered by genetically engineered NP cells, on immunohistochemical MMP/ADAMTS expression after 48 hours (five IVDs) and two weeks (three IVDs).

Adenoviral constructs incorporating green fluorescent protein (Ad-GFP; control) or IL-1Ra were generated by Cre-lox recombination [15]. Viruses grown in HEK 293 cells were purified using CsCl density gradient purification. IVD cell monolayers transfected at an MOI (mean number of viruses per target cell) of 300 were cultured overnight at 37°C in 5% CO_2_. Viable monolayer cells were suspended, centrifuged at 1500 g for 10 minutes and resuspended at a density of 100,000 cells per 20 μl.

After 24 hours in standard medium, encapsulated explants were injected with either 20 μl of medium, or 20 μl of medium containing transfected cells or untransfected control cells. IL-1Ra was measured by ELISA. (Transfected cells produced 100 to 200 ng/ml per explant.) After a further 48 hours, 5 blocks, and after 14 days another 3 blocks, were fixed in formalin and paraffin embedded.

IHC was used to stain 4 μm sections for MMP1, -3, -7, and -13 and ADAMTS4. The IHC techniques were as described above for MMP3, -7 and -13. Monoclonal mouse anti-human MMP1 antibody was obtained from R&D systems and polyclonal goat anti-human ADAMTS4 from Santa Cruz, Santa Cruz, California, USA. Chymotrypsin antigen retrieval was required for anti-MMP1. Positive cells were expressed as a percentage of all nucleated cells.

### Statistical analysis

Between group differences were assessed using a two-tailed ANOVA analysis.

## Results

### *In situ *hybridization

All the tissue used showed evidence of an expected degree of cell viability. In the normal tissue this was >95% of cells reacting for polyadenylated RNA. Because of apoptosis a proportion of cells in degenerate IVDs will be unreactive; in these IVDs >70% (range 71% to 94%) of cells were positive.

### *In situ *zymographic studies

Matrix degradation occurred below cell profiles rather than intercellular matrix.

#### Cytokine and anti-cytokine effects

**Figure 1:**
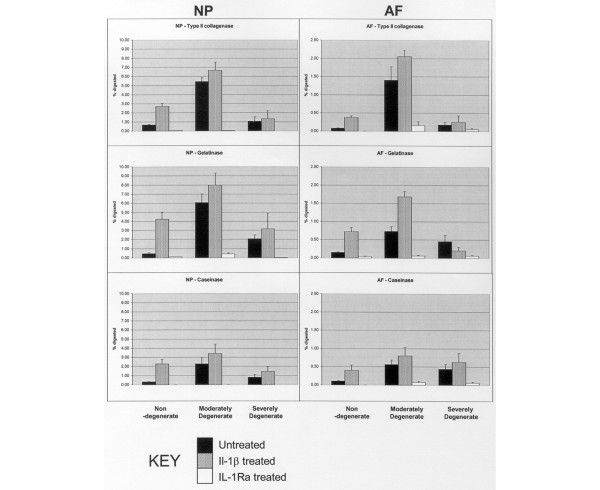
Effects of IL-1 and IL-1 receptor antagonist (IL-1Ra) on the *in situ *zymography profile of degenerate and non-degenerate intervertebral discs (IVDs). The six graphs compare matrix degrading activity in the nucleus pulposus (NP) and annulus fibrosus (AF) for the three substrates in non-degenerate, moderately degenerate and severely degenerate IVDs and following treatement with IL-1 and IL-1Ra. The data are expressed in terms of absolute area of gel degraded (Y-axis = area degraded (maximum 10% in NP and 2.5% in AF)). Data are expressed as mean + standard error of the mean.

**Figure 2:**
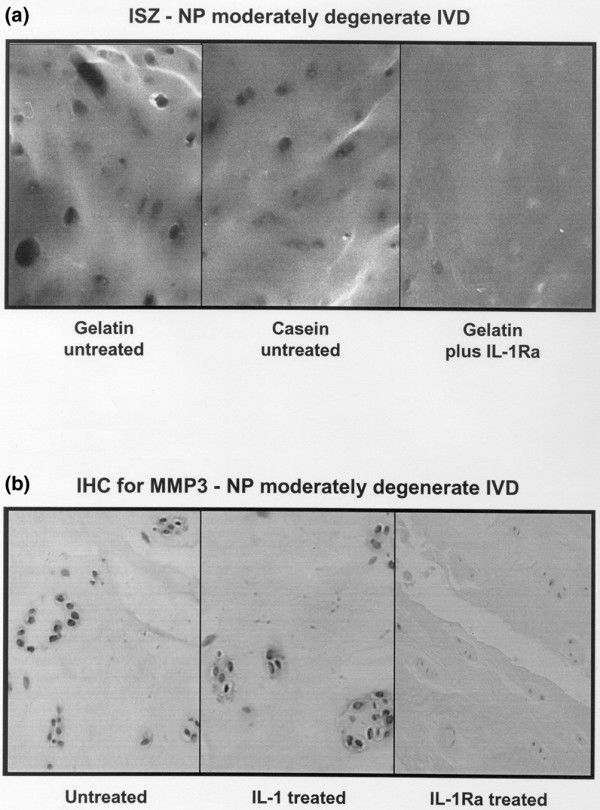
Images illustrating the histological appearances of *in situ *zymography (ISZ) and immunohistochemistry (IHC) in moderately degenerate intervertebral discs (IVDs). **(a) **Photomicrographs taken using the fluorescent microscope. The black rounded areas are regions of matrix degradation. There are none of these areas within the right hand image of a specimen treated with IL-1 receptor antagonist (IL-1Ra). The outlines of cells can just be made out in this image as pale grey ghosts. **(b) **IHC performed on formalin fixed, paraffin embedded tissue derived from the ISZ experiments. Matrix metalloproteinase (MMP)3 is seen in the cytoplasm of many of the cells in the untreated tissue. It increases (non-significantly) after IL-1 treatment and is markedly reduced following IL-1Ra treatment (right-hand image). In this image the nuclei of the nucleus pulposus (NP) cells can be seen where they have taken up the counterstain, which contrasts with the darker cytoplasmic staining of IHC reaction product for MMP3 in the left and center images.

ISZ data for untreated and IL-1/IL-1Ra treated tissue are shown in Figure 1 and illustrated in Figure 2a.

##### Non-degenerate IVDs

Type II collagenase, gelatinase and caseinase activity were present in NP and AF. IL-1β increased the degradation area of all three substrates by four- to ten-fold depending on enzyme and region of the IVD (greatest, gelatinase in the NP; least, caseinase in the AF). IL-1Ra eliminated 95% to 100% of activity of all enzymes in the AF and NP (*p *< 0.01).

##### Moderately degenerate IVDs

Enzyme activity was present against all 3 substrates, 4- to 13-fold higher than in the non-degenerate IVDs, and highest in the NP. IL-1β increased degradation of all three substrates, but with the exception of gelatinase activity in the AF (*p *< 0.05), this did not reach statistical significance. IL-1Ra eliminated 95% to 100% of all enzyme activity (*p *< 0.01).

##### Severely degenerate IVDs

Degradation of all three substrates was greater in severely degenerate than non-degenerate IVDs (*p *< 0.01), but lower than in moderately degenerate IVDs (*p *< 0.01). The pattern of IL-1 and IL-1Ra effects was similar to that in moderately degenerate IVDs, except in the AF, where IL-1Ra decreased caseinase activity by 85%, gelatinase activity by 65% and collagenase activity by 50%.

#### Enzyme inhibitors

**Figure 3:**
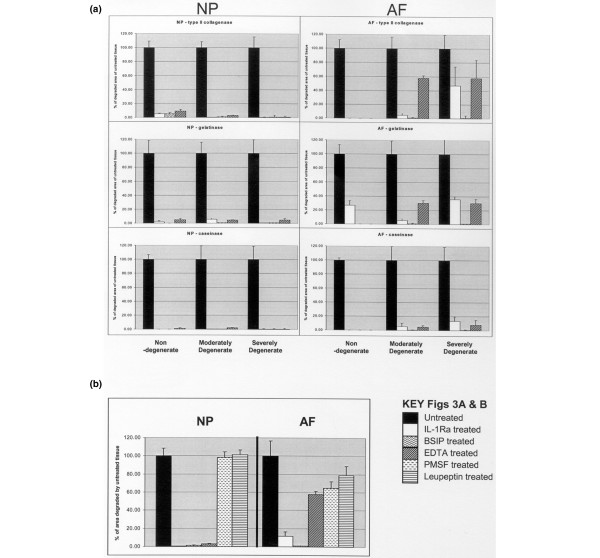
Effects of enzyme inhibitors on matrix degradation. All results are expressed as a percentage of the mean of the measured area of degradation of the untreated samples. All data given as mean ± standard error of the mean. **(a) **Comparison of the effects of IL-1 receptor antagonist (IL-1Ra), broad spectrum inhibitor of proteases (BSIP) and EDTA on degradation of the three matrices in non-degenerate, moderately degenerate and severely degenerate intervertebral discs (IVDs). In the nucleus pulposus (NP) all the inhibitors effectively eliminate enzyme activity. In the annulus fibrosus (AF) of moderately degenerate IVDs, BSIP and IL-1Ra eliminate enzyme activity but EDTA only reduces type II collagenase and gelatinase activity. In the AF of severely degenerate IVDs, BSIP eliminates collagenase and gelatinase activities, but both IL-1Ra and EDTA only reduce them. **(b) **Comparison of the effects in AF and NP of moderately degenerate IVDs of all the inhibitors employed in this study (IL-1Ra, BSIP, EDTA, phenyl methyl sulphonyl fluoride (PMSF) and leupeptin) to show differences in the enzyme subgroups involved. The key finding is in the AF, where IL-1Ra eliminated all enzyme activity but EDTA did not, indicating that the enzymes involved were driven by IL-1 but were not metal-dependent proteases (MDPs). By contrast with the NP, PMSF and leupeptin did reduce enzyme activity, indicating that those IL-1 driven, non-MDP enzymes were serine/cysteine proteases.

BSIP eliminated all enzyme activity in every situation. Where enzyme activity was present, EDTA caused >95% decrease in all enzyme activity in the NP and AF (Figure 3a), with the exception of the AF of moderately and severely degenerate IVDs, where it inhibited all caseinase activity (*p *< 0.01) but only 40% of type II collagenase (*p *< 0.02) and 70% of gelatinase (*p *< 0.02) activity. Here, PMSF caused a 35% reduction in collagenase and 22% reduction in gelatinase activity (*p *< 0.05 for both) and leupeptin a 21% reduction in collagenase activity (*p *< 0.05), indicating serine/cysteine protease activity in the AF. This is illustrated in Figure 3b, which documents graphically the effects of IL-1Ra, BSIP, EDTA, PMSF and leupeptin on collagen type II matrix degradation in the NP and AF. The other inhibitors had no effect (data not shown).

#### Cadaveric versus live tissue

There were no statistical differences in any experiment between cadaveric and surgically excised IVDs matched for age, sex or degradation score.

### Immunohistochemical analysis of treated tissue blocks

Immunohistochemical data are shown in Table 2 and illustrated in Figure 2b. Data are only shown for untreated and IL-1β/IL-1Ra-treated samples as no other treatment affected enzyme expression.

**Table 2:** Percentage of cells expressing IHC detectable MMP3, -7 or -13 following incubation of tissue in standard medium, or medium supplemented with IL-1 or IL-1Ra for 48 hours

			MMP3		MMP7		MMP13	
					
			Mean (percent)	SEM	Mean (percent)	SEM	Mean (percent)	SEM
**Non-degenerate**	NP	Untreated	0.9	1.0	4.2	2.1	2.7	0.9
		IL-1	6.5	1.2	35.8	7.6	11.3	5.2
		IL-1Ra	0	0.0	0.2	0.1	0.2	0.1
	AF	Untreated	0.3	0.2	0.4	0.2	0.3	0.2
		IL-1	1.2	0.5	1.9	0.9	1.5	0.5
		IL-1Ra	0.0	0.0	0.2	0.1	0.0	0.0
**Moderately degenerate**	NP	Untreated	10.2	4.1	72.6	10.9	31.9	7.7
		IL-1	14.1	5.6	83.4	10.1	38.2	10.7
		IL-1Ra	0.0	0.0	3.3	1.2	2.7	1.1
	AF	Untreated	1.9	0.9	3.5	1.1	1.0	0.4
		IL-1	2.8	1.4	5.1	0.9	1.5	0.5
		IL-1Ra	0.2	0.1	0.2	0.1	0.1	0.1
**Severely degenerate**	NP	Untreated	3.6	1.2	17.9	6.0	16.2	7.2
		IL-1	6.5	3.1	19.4	6.5	19.9	6.3
		IL-1Ra	0.2	0.1	2.2	1.1	2.1	1.2
	AF	Untreated	2.0	0.4	0.9	0.4	0.2	0.1
		IL-1	2.8	0.7	1.1	0.2	2.3	0.7
		IL-1Ra	0.2	0.1	0.2	0.1	0.1	0.0

AF, annulus fibrosus; IHC, immunohistochemistry; IL-1Ra, interleukin-1 receptor antagonist; MMP, matrix metalloproteinase; NP, nucleus pulposus; SEM, standard error of the mean.

#### Non-degenerate tissue

The 3 MMPs were expressed by 1% to 4% of NP cells and 0.3% to 1% of AF cells. Enzyme expression in both regions increased four- to ten-fold with IL-1 treatment (*p *< 0.01). IL-1Ra reduced immunodetectable enzyme expression by at least 90% for all enzymes in AF and NP (*p *< 0.02).

#### Moderately degenerate IVDs

When compared with non-degenerate tissue, cells within these IVDs showed significantly greater (*p *< 0.01) innate expression of all three enzymes. IL-1 increased enzyme expression generally but only MMP3 in the NP significantly (*p *< 0.05), and MMP7/MMP13 (*p *< 0.05) in the AF. In AF and NP, IL-1Ra reduced enzyme expression by at least 90% (*p *< 0.01).

#### Severely degenerate IVDs

Expression of MMP3, -7 and -13 was significantly greater than in non-degenerate NP (*p *< 0.02) but not the AF. IL-1β increased and IL-1Ra decreased enzyme expression in a similar pattern to that in moderately degenerate IVDs.

### Correlation of enzyme activity and protein expression

To assess any relationship between matrix degradation (ISZ) and MDP expression (IHC), scatter plot analyses was performed. These show statistically significant correlations (*p *< 0.01) for type II collagenase activity versus MMP13 expression, gelatinase and caseinase activity versus MMP7 expression, and caseinase and gelatinase activity versus MMP3 expression. Other combinations did not reach statistical significance.

### Effects of IL-1Ra gene therapy on enzyme expression

**Figure 4:**
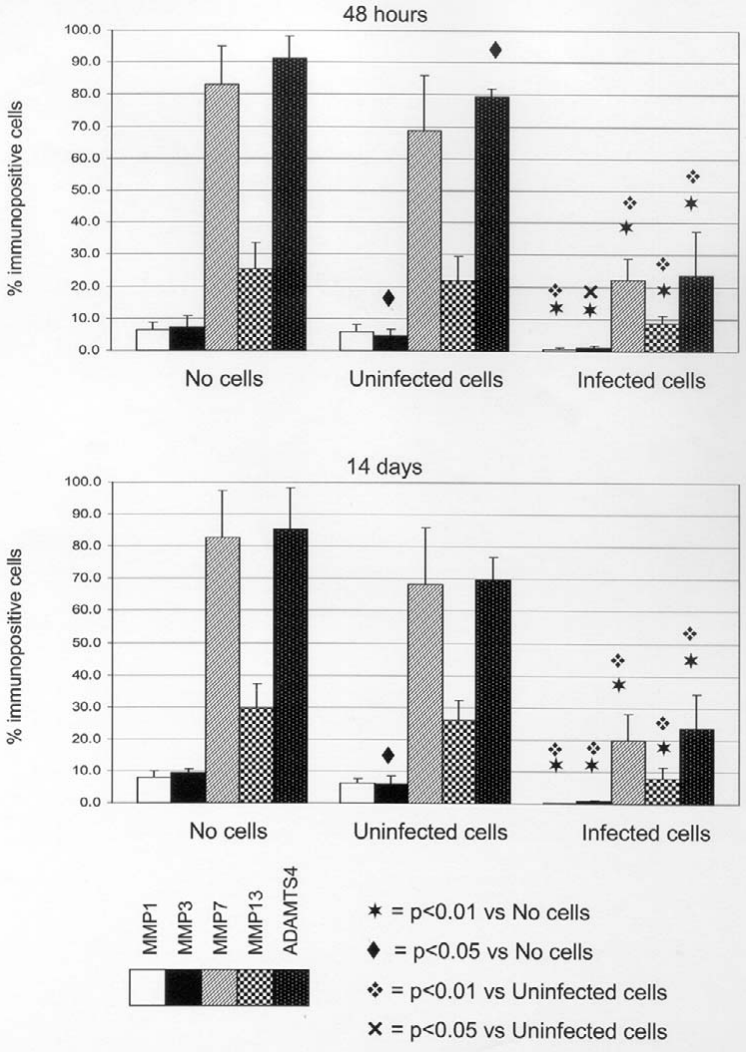
The effect of IL-1 receptor antagonist (IL-1Ra)-transfected cells on matrix metalloproteinase (MMP) and a disintegrin and metalloproteinase with thrombospondin motifs (ADAMTS) expression in moderately degenerate intervertebral discs (IVDs). The graphs show the effects of medium alone, untransfected cells and cells transfected with IL-1Ra on the proportion of nucleus pulposus (NP) cells expressing the various enzymes by NP cells in human IVD explants at 48 hours and 14 days after injection of medium, medium containing non-transfected cells and medium containing transfected cells. Data expressed as mean percent + standard error of the mean.

The effects of IL-1Ra delivered by transfected cells on MMP/ADAMTS expression in moderately degenerate NP are shown in Figure 4 (Ad-GFP data are not shown as they are identical to untransfected cells).

Medium had no effect on enzyme expression. Injection of untransfected/Ad-GFP transfected NP cells into degenerate NP explants led to a reduction in the percentage of cells expressing all enzymes at 48 hours (statistically significant for only MMP3 (*p *< 0.05) and ADAMTS4 (*p *< 0.05)) and 2 weeks (significant for MMP3 only (*p *< 0.05)).

Injection of cells expressing IL-1Ra caused a dramatic and statistically significant (*p *< 0.01) reduction in expression of all enzymes at 48 hours. This was sustained for two weeks after injection with no statistical difference in expression over that period.

## Discussion

In DIVD, reduction in vertebral separation secondary to enzyme-mediated matrix loss has been causally linked to CLBP, a debilitating and economically important disorder [27,28] for which current treatments have only limited success. Understanding the molecular mechanisms leading to matrix degradation is a key step in designing novel treatments for degeneration [29].

It is known from expression studies that in degenerate human IVDs, key MDPs, notably MMP1, -3, -7 and -13, and ADAMTS4, are upregulated [5,30,31]. In three-dimensional alginate culture, IL-1β upregulates expression of these enzymes by IVD cells [15]. In non-degenerate IVD, cells express IL-1α/β [14], expression matched by that of IL-1Ra. In degeneration, however, IL-1α/β are upregulated without increased IL-1Ra. Co-culture of cells from degenerate IVD with cells engineered to over-express IL-1Ra inhibits IL-1 synthesis [15]. These and similar data derived from human gene/gene product expression, experimental animal and cell-based *in vitro *studies form the basis of proposed mechanism for matrix loss in the degenerate IVD in which matrix degradation is driven by IL-1 and mediated through MDPs. As the biology of MDP and IL-1 are well known and naturally occurring inhibitors (TIMPs, IL-1Ra) are already used to treat joint disease, MDP and IL-1 are attractive therapeutic molecular targets in the setting of degeneration of the IVD and discogenic low back pain [16].

In the current study we have examined the evidence for the existence of this mechanism in intact human IVD tissue and the potential for MDP inhibitors and IL-1Ra to reverse the disease process. The novel data we are reporting are: direct evidence for MDPs being the major enzymes involved in matrix degradation in the NP, but serine/cysteine proteases having an important role with MDPs in the AF; IL-1Ra and EDTA inhibiting type II collagnease, caseinase and gelatinase activity in the NP; EDTA having limited effects on matrix degrading activity in the AF whereas IL-1Ra almost completely eliminates it; matrix degrading activity correlates with MDP expression in human IVD; IL-1Ra delivered by gene therapy can significantly diminish MDP expression for sustained periods.

Our data show that MDPs are the major functional enzyme group in non-degenerate and degenerate IVD. Although this is the first study to demonstrate this directly, associations between enzyme expression and the extent of degenerative changes [5,6,32] or duration of symptoms [33] have indicated this should be the case. Expression studies of MMP1, -2, -3, -7, -8, -9, -13 and -19, and ADAMTS 4 and -5 in DIVD [5,6,8,14,30-39] have particularly implicated MMP1, -3, -7 and -13, and ADAMTS4 in matrix degradation.

Most previous studies of human IVD have studied herniated tissue; however, herniated tissue is not believed to be the seat of the processes of degeneration. By selecting cases for study in which whole IVDs have been excised and by using quantitative image analysis, we have been able to investigate disease mechanisms within those IVD components primarily affected by the processes of degeneration, localize matrix degrading activity within the NP and AF, and correlate matrix degrading activity with enzyme expression. This has shown that MDPs are the key mediators of matrix degradation in the NP, but that matrix degradation in the AF is mediated by both MDPs and serine/cysteine proteases. In this context it is of interest that the serine/cysteine proteases cathepsins G and L [7,9] have been identified in human AF. Our data have also shown that, in keeping with these data, the MDP inhibitor EDTA eliminated all enzyme activity in the NP, but only part of that in the AF. By contrast, IL-1Ra gave ≥95% inhibition of enzyme activity in both. Furthermore, it inhibited enzyme expression when EDTA did not, suggesting IL-1 would be a better clinical target than MDP in the management of DIVD.

The first evidence that IL-1 might be involved in matrix degredation in DIVD came in 1988 from *in vitro *experiments employing rabbit AF cells [40]. In 1997, a ground-breaking study [36] extended these data into human IVD but did not fully localize enzymes within the IVD, relate enzyme activity to expression or show the inhibitory potential of IL-1Ra.

Demonstrating that inhibitors of IL-1Ra almost completely eliminate matrix degrading activity places IL-1 as a key regulator of matrix degradation in DIVD. This would also explain why IL-1 has greater expression in those IVDs demonstrated clinically to be the source of CLBP [14,41,42] and the links between back pain and possession of IL-1aT889, IL-1RNA1812, and IL-1bT3954 alleles [43-45]. That IL-1 upregulates its own expression [14] is reflected in the data (Figure 4). These show IL-1Ra quickly extinguishes MDP expression but, in the absence of IL-1Ra, IL-1-driven MDP expression is maintained even in prolonged tissue culture outside the disease context of the spine. Breaking the auto-stimulation of IL-1 production could be key to inhibiting progression of degeneration.

Probing IVD samples with IHC and ISZ has enabled the degrading activity of specific substrates to be related to expression of particular MMPs. We have shown a statistically significant relationship between these two variables. This proved important in the last part of this study. Maintaining adequate tissue levels of IL-1Ra in IVDs could be problematic. The avascular IVD does not lend itself to delivery of drugs/biomolecular regulators through the circulation, nor is repeated direct injection practical. To identify and test a suitable method for delivering IL-1Ra long-term to the IVD, we have explored the use of cells engineered to over-express IL-1Ra. It was hypothesized in 1997 [46] that gene therapy might be an effective method for delivering disease modifying biologicals to the IVD, but its use has not been previously reported in human IVD tissue. The 100 to 200 ng/ml of IL-1Ra produced by transfected cells significantly decreased the proportion of cells expressing 5 key degradative enzymes 48 hours after injection of the cells, an effect sustained for 2 weeks without evidence of reduced efficacy.

Injection of untransfected and Ad-GFP transfected NP cells appeared to have some effect on enzyme expression. We have previously shown that untransfected cells secrete picogram quantities of IL-1Ra, which could account for some of the changes [15]. However, it is more likely that this is an effect of the method used to assess therapeutic effectiveness, the introduced cells contributing to the total cell pool, but not the enzyme immunoreactive population.

To the best of our knowledge this is the first published evidence that gene therapy delivered into human IVD explants can change the molecular mechanisms underlying degeneration. However, many problems still need to be addressed before this can be a practical proposition, key amongst which are ensuring that transfected cells can continue to synthesize active product within the environment of the degenerate human IVD [47]. Thus, whilst experimental data have shown that direct injection of viral vector into normal rabbit IVDs leads to synthesis of gene product and increased production of proteoglycans [48], the increased nutrient and oxygen transfer into IVD tissue, the cytokine and eicosanoid load and the growth factor environment of normal animal IVDs are markedly different to those of degenerate human IVDs. By using intact human IVDs and controlled culture conditions we have begun to address this by reproducing the *in vivo *situation more closely than has been possible previously.

## Conclusion

This study has built on previous, largely observational data implicating IL-1 in the matrix degradation that characterizes degeneration of the IVD. Specifically, we have shown for the first time that: native cells in intact IVD tissue produce gelatin, casein and type II collagen degrading enzymes; IL-1 delivered to non-degenerate IVDs will induce expression of these enzymes; and IL-1Ra delivered directly to explants of degenerate IVDs will almost completely eliminate this enzyme activity. A further key and novel observation has been to show that the expression of specific matrix degrading enzymes is proportional to matrix degrading activity.

Finally, we have shown that IL-1Ra can be effectively delivered to degenerate IVDs by injecting cells genetically engineered to overexpress IL-1Ra into IVD tissue, where it will significantly inhibit expression of enzymes implicated in degradation of the IVD. Such an approach applied *in vivo *could form the basis either of a therapy for preventing matrix loss in early degeneration or in preparing the diseased IVD for tissue engineering or regenerative medicine strategies designed to restore a normal matrix [49,50].

## Abbreviations

ADAMTS = a disintegrin and metalloproteinase with thrombospondin motifs; Ad-GFP = adenoviral constructs incorporating green fluorescent protein; AF = annulus fibrosus; ANOVA = Analysis of variables; BSIP = broad spectrum inhibitor of proteinases; CcCl = Cesium chloride; CLBP = chronic low back pain with or without sciatica; DIVD = degeneration of the intervertebral disc; GFP = green fluorescent protein; IHC = immunohistochemistry; IL = interleukin; IL-1Ra = interleukin-1 receptor antagonist; ISZ = in situ zymography; IVD = intervertebral disc; MDP = metal-dependent proteases; MMP = matrix metalloproteinase; MOI = Multiplicity of infection; mRNA = messenger ribose nucleic acid; NP = nucleus pulposus; PMSF = phenyl methyl sulphonyl fluoride; RNA = ribose nucleic acid; T = thymidine.

## Competing interests

The authors declare that they have no competing interests.

## Authors' contributions

CLM performed the gene delivery studies and generated and analyzed the data from these experiments. JAH together with AJF conceived the study, participated in its design, co-ordination, analysis and interpretation, and helped to draft the manuscript. AJF together with JAH developed the ISZ techniques, performed and analyzed the ISZ studies, participated in the design of the overall study and were responsible for producing the final manuscript. All authors read and approved the final manuscript.
